# Editorial: Global excellence in inflammation pharmacology

**DOI:** 10.3389/fphar.2023.1307503

**Published:** 2023-10-17

**Authors:** Sukanya Saha, Shatadal Ghosh

**Affiliations:** ^1^ Neurobiology Laboratory, Department of Health and Human Services, National Institute of Environmental Health Sciences, National Institutes of Health, Durham, NC, United States; ^2^ Division of Hematology and Blood Research Center, Department of Medicine, University of North Carolina at Chapel Hill, Chapel Hill, NC, United States

**Keywords:** inflammation, pharmacological interventions, global alliance, therapeutics, diseases

In our day-to-day life, acute inflammation occurs commonly as a part of the body’s regular healing process post an injury or infection and is usually short term in nature. The problem arises when this inflammation becomes chronic, due to the failure in resolving itself, leading to a spectrum of diseases which explicitly contributes to more than 50% of worldwide mortality (GBD, 2017 Causes of Death Collaborators, 2018; Furman et al., 2019) Thus, prolonged inflammation is emerging as a serious threat to the global population and socioeconomic sustainability. Advancements in effective anti-inflammatory therapies have been significantly evolving, but challenges persist (Netea et al., 2017). Hence, scientists globally or in global alliance, with varied scientific perspectives, are actively working on finding out pharmacological interventions against inflammation associated pathogenesis and diseases. A major part of the research is also focused on scientific advancements of evolving therapeutic strategies by identifying the central signaling molecules or cascades involved in the onset and progression of chronic inflammation. This special edition Research Topic *Global Excellence in Inflammation Pharmacology* aims in emphasizing on the recent progress made in these fields, highlighting the diversified research performed across the entire breadth of Inflammation Pharmacology and providing insights to it. This Research Topic comprises of four extensive literature reviews discussing the potential pharmacological interventions and their allied risk in inflammation associated diseases.

One of the prevalent inflammatory diseases with approximately 500 million people suffering worldwide, is osteoarthritis (OA). OA involves joint inflammation and is degenerative in nature (Hunter et al., 2020). Literature suggests that peroxisome proliferator-activated receptor γ coactivator 1α (PGC-1α), the mitochondrial biogenesis master regulator, is downregulated in OA by molecules like AGE. The review article by Wang et al. discusses the chondroprotective role of PGC-1α in OA and provides an understanding of its mechanistic regulation by pathways like AMPK/SIRT1, SIRT3, mTOR, etc., in delaying OA onset and progression. They elaborate how PGC-1α inhibits apoptosis and alters mitochondrial biogenesis, providing protection to the chondrocytes. They also discuss reports on PGC-1α modulating natural compounds, chemical drugs and novel nanohybrid formulations for the treatment of OA. This review sheds light on the up-to-date developments of therapeutic strategies for OA along with providing a detailed understanding on the pathogenesis of OA.

Another article by Huang et al. reviews the therapeutic potential of natural bioactive compound Chlorogenic acid and how it acts as a protective molecule against inflammation driven conditions such as cardio-cerebrovascular pathology and diabetes mellitus. It exerts anti-inflammatory potential by the inhibition of pro-inflammatory cytokines, prostaglandin E2 and nitric oxide along with modulation of key cellular signaling pathways like NF-κB, MAPK and Nrf2. They further discuss the limitation of the use of chlorogenic acid due to its inherent low bioavailability and how this is overcome by upgraded delivery systems, positively regulating the release mechanisms, stability, and bioactivity of Chlorogenic acid, making it a more promising pharmacologically active anti-inflammatory intervention.

“Metabolic syndrome,” a relatively newly recognized pathology, primarily comprises of different detrimental inflammatory conditions of the body that leads to hypertension, hyperglycemia, insulin resistance, obesity, hypercholesterolemia, and so on. This is intricately associated with global health risk, precisely increasing development of cardiovascular and atherosclerotic diseases. In their thorough review article, Collotta et al. discuss in detail about the current advancements of Janus Kinase (JAK) inhibitors for their efficacy in treating metabolic syndrome, highlighting the importance of JAK-STAT signaling pathways in such chronic inflammatory conditions.

Continuing with the discussion of the JAK inhibitors in inflammatory diseases, in their systemic review, Xu et al. extensively discuss about the meta-analysis on the efficacy of JAK inhibitors in immune-mediated inflammatory diseases (IMID) and the side effects reported such as higher risk of herpes zoster infection in patients associated with the use of JAK inhibitors. Herpes zoster (or shingles) is a viral infection caused by the reactivation of the varicella-zoster virus in patients who have a history of chickenpox infection. Their study indicates that except for a few JAK inhibitors, like peficitinib, baricitinib, and tofacitinib, there is no association of higher risk of herpes zoster infection in patients with IMIDs.
